# Efficacy of high-dose rosuvastatin preloading in patients undergoing percutaneous coronary intervention: a meta-analysis of fourteen randomized controlled trials

**DOI:** 10.1186/s12944-015-0095-1

**Published:** 2015-08-27

**Authors:** Yilong Pan, Yuan Tan, Bin Li, Xiaodong Li

**Affiliations:** Department of Cardiology, Shengjing Hospital of China Medical University, Shenyang, China; Department of Anesthsia, Shengjing Hospital of China Medical University, Shenyang, China

**Keywords:** Rosuvastatin, Clinical events, Peri-procedural myocardial infarction, Meta-analysis

## Abstract

**Background:**

Numerous studies have evidenced that statins can reduce the incidence of cardiovascular disease. However, the effects of high-dose rosuvastatin (RSV) preloading in patients undergoing percutaneous coronary intervention (PCI) are controversial.

**Objective:**

We attempted to identify and quantify the potential cardioprotective benefits of high-dose RSV preloading on final thrombolysis in myocardial infarction (TIMI) flow grade, major adverse cardiac events (MACE), and peri-procedural myocardial injury (PMI) in patients undergoing PCI.

**Methods:**

Pubmed, EMBASE, Cochrane Central Register of Controlled Trials and ISI Web of Science databases were systematically searched for randomized controlled trials (RCTs) up to June 2015. We assessed the incidence of MACE and PMI in all enrolled patients for subgroups stratified by clinical presentation and previous statin therapy during the follow-up period.

**Results:**

Fourteen trials with 3368 individuals were included in our meta-analysis. High-dose RSV preloading before PCI lead to a 58 % reduction in MACE (odds ratio [OR] = 0.42, 95 % confidence intervals [CI]: 0.29-0.61, *P* < 0.00001) and a 60 % reduction in PMI (OR = 0.40, 95 % CI: 0.25–0.63, *P* < 0.0001). This procedure also improved the final TIMI flow grade in patients undergoing PCI (OR = 1.61, 95 % CI: 1.09–2.38, *P* = 0.02). The benefits on MACE were significant for both stable angina patients (OR = 0.42, 95 % CI: 0.21-0.87, *P* = 0.02) and acute coronary syndrome (ACS) patients (OR = 0.42, 95 % CI: 0.27-0.65, *P* < 0.0001); and for both statin naïve patients (OR = 0.42, 95 % CI: 0.28-0.64, *P* < 0.0001) and previous statin therapy patients (OR = 0.28, 95 % CI: 0.10-0.73, *P* = 0.01).

**Conclusion:**

High-dose RSV preloading can significantly improve myocardial perfusion and reduce both MACE and PMI in patients undergoing PCI. The cardioprotective benefits of RSV preloading were significant in not only stable angina and ACS patients but also statin naïve and previous statin therapy patients. The cardioprotective benefits of RSV preloading in the follow-up period mainly resulted from a reduction in spontaneous MI and TVR, especially for ACS and statin naïve patients.

## Introduction

Percutaneous coronary intervention (PCI) is extensively used as a reperfusion strategy for coronary artery disease. Although this procedure is relatively safe and the procedure-related complications have markedly decreased with years, peri-procedural myocardial injury (PMI) can still occur [1]. The most common mechanism is side-branch occlusion during PCI, and distal embolism, coronary dissection, and inflammation can also result in PMI. Moreover, PMI lead to an increased incidence of death at follow-up (hazard ratio[HR] = 1.2, 95 % CI: 1.04-1.39) after adjustment for baseline covariates [2]. Research previously focused on the improvement of antithrombotic agents and vasodilators in decreasing the incidence of cardiac ischemic events during PCI, while recently it was found that pretreatment with statins may significantly reduce major adverse cardiac events (MACE) and PMI in patients undergoing PCI [3, 4]. It has already been proved in some meta-analyses of randomized controlled trials (RCTs) [5, 6]. A meta-analysis of 3341 individuals from 13 randomized studies suggested that statin preloading leads to a significant decrease in 30-day MACE and PMI in patients undergoing PCI [5]. However, this research was not able to indicate whether statin pretreatment was effective in stable angina or acute coronary syndrome (ACS) patients, as patients were not assigned to subgroups according to their clinical presentation. Another updated meta-analysis comprising 5526 patients from 24 RCTs indicated that the cardioprotective benefits of statin preloading on MACE were effective for statin naïve or ACS patients [7]. However, recent published trials in which patients received high-dose rosuvastatin treatment prior to PCI were not included in this study, and this may have influenced the final clinical outcomes.

Rosuvastatin (RSV), an inhibitor of 3-hydroxy-3-methylglutaryl coenzyme A (HMG-CoA) reductase, has a number of pleiotropic effects, including antioxidative, antithrombotic, anti-inflammatory, and cardiovascular protective outcomes. Recently, investigators found a lower rate of MACE and PMI when patients undergoing PCI received high-dose RSV pretreatment [8–21]. However, most of the prospective trials lacked the power to detect differences from clinical outcomes, due to small size, varying endpoint definitions, individuals with different clinical presentation, and diverse regimens of RSV therapy. Therefore, we systematically evaluated the clinical benefits of high-dose RSV preloading prior to PCI by conducting a meta-analysis including all relevant RCTs.

## Methods

### Search strategy

In this study, we searched PubMed, EMBASE, the Cochrane Central Register of Controlled Trials and ISI Web of Science databases up to June 2015 to determine prospective RCTs comparing the cardiovascular events of RSV preloading with control (placebo, no-statin, or current statin therapy) in patients undergoing PCI. Key words for searching were: “rosuvastatin”, “percutaneous coronary intervention”, “PCI”, “stents”, “angioplasty”, “randomized” and “randomly”. We also screened previous meta-analyses and the references of selected studies. No language restrictions were used.

### Study selection

Trials were included if: (1) they were RCTs involving humans; (2) they selected patients with ACS (ST-segment elevation myocardial infarction [STEMI], unstable angina pectoris, or non-ST-segment elevation myocardial infarction [NSTEMI]) or stable angina; (3) they enrolled patients who were statin naïve or undergoing current therapy with statins; (4) they reported information on MACE including mortality, spontaneous myocardial infarction (MI), and target vessel revascularization (TVR) as well as PMI after PCI. Thrombolysis in myocardial infarction (TIMI) classification post-PCI was also recorded. Exclusion criteria included: (1) non-randomized controlled trials; (2) no outcomes of interest; (3) duplicate reports without additional or updated outcome data.

### Data extraction

Two authors (YLP and YT) extracted the data in all the included trials, and any disagreement was resolved by discussion with the third author (BL). Firstly, the following information was extracted from each study: the first author’s name, year of publication, sample size of the trial, type of population, clinical features, regimen of statins before and after PCI, study duration, and definition of PMI. Furthermore, we extracted the baseline characteristics and procedural details in all enrolled patients. Finally, we extracted data according to the clinical outcomes including mortality, spontaneous MI, TVR, and overall MACE during the follow-up period in each group.

### Quality assessment

Study quality was evaluated based on the quality assessment criteria for RCTs in the Cochrane Handbook for Systematic Reviews of Interventions, including random sequence generation, allocation concealment, blinding of investigators, participants and outcome assessors, incomplete outcome data, selective reporting and other sources of bias.

### End-Points

The primary end-points were MACE and PMI. We used the PMI definition which was taken from the original articles. Secondary end-point was final TIMI flow grade, which assessed myocardial perfusion after PCI. We carried out sub-group analyses according to the clinical condition (ACS and stable angina) or current statin therapy (statin naïve and previous statin therapy).

### Statistics

Odds ratio (OR) with 95 % confidence intervals (CI) was used to express dichotomous variables, such as the incidence of PMI, MACE, and post-PCI TIMI flow 3. Heterogeneity among studies was quantified using the *I*^2^ statistic, defined as *I*^2^ > 50 %. In that case, the random effects model was used; otherwise, the fixed effect model was chosen. Funnel plots and Egger’s regression test were used to illustrate the potential publication bias. Results were considered statistically significant at *P* < 0.05. RevMan 5.3 software (Cochrane Collaboration, Copenhagen, Denmark) was used for statistical analysis.

## Results

### Selected studies and baseline characteristics

**Fig. 1:**
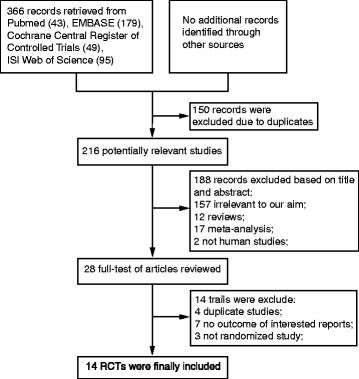
Flow diagram of study selection process. RCTs, randomized controlled trials

Based on the search strategy described above, 366 potentially relevant studies were initially included by titles and abstracts (Fig. 1), and 28 publications were selected. After reading the full texts of these publications, fourteen further studies were excluded as they were either duplicate studies, no outcome of interest reported, or were non-randomized studies. Finally, 3273 individuals from fourteen RCTs [8–21] were included, of which 1671 were randomized to the high-dose RSV group and 1602 were randomized to control.

Table 1 shows the baseline characteristics of all the studies, which were published between 2010 and 2014. There were ten trials [8, 10, 11, 13, 15, 17–21] with only statin naïve patients, one trial [16] with only previous statin therapy patients, and three trials [9, 12, 14] with both of two. Five trials [9, 14–16, 21] included stable angina patients, six trials [8, 11, 13, 17, 19, 20] included NSTE-ACS patients, two trials [12, 18] included STEMI patients, and one trial [10] included ACS patients. Thirteen trials [8–13, 15–21] included short-term RSV pretreatment (immediate to 24 h), while one trial [14] included relatively long-term RSV pretreatment (5 to 7 days). Other characteristics of the included studies were follow-up duration and definition of PMI. The baseline characteristics of the patients and the procedural details in all trials are shown in Table 2. Approximately two-thirds of the overall population were male, three-fifths of those had hypertension, and one-third of those had diabetes. Preprocedural aspirin and clopidogrel were given to patients in all studies, and patients among four trials [11, 12, 15, 16] received a loading dose of clopidogrel (600 mg), while the number of patients treated with clopidogrel in one trial [10] were unclear. Patients in a total of 11 trials [8, 11–13, 15–21] received glycoprotein IIb/IIIa inhibitors (GPIs) at the operators’ discretion, while the remaining trials [9, 10, 14] did not mention the use of GPIs. Almost four-fifths of the patients in seven trials [8, 10, 13, 15, 17, 20, 21] underwent implantation of drug-eluting stents.

**Table 1:** Characteristics of the included studies

Study	Patients (RSV/ Con)	Type of population	Clinical feature	Timing before PCI	Regimen after PCI	Follow-up	Definition of PMI
Gao [19]	59/58	Statin naïve	NSTE-ACS	RSV 20 mg 12 h and 10 mg 2 h before PCI VS placebo treatment	RSV 10 mg/d for at least 1 year	6 months	CKMB > 3 UNL
Li [18]	103/ 100	Statin naïve	STEMI	RSV 20 mg before PCI VS RSV 10 mg treatment	RSV 10 mg/d for 3 months	30 days	CKMB > 3 UNL
Luo [17]	31/36	Statin naïve	NSTE-ACS	RSV 20 mg 12 h and 20 mg 2 h before PCI VS no statin pretreatment	RSV 10 mg/d	30 days	cTnI > 3 UNL
Takano [14]	104/ 106	Mixed	Stable Angina	RSV 20 mg 5-7day before PCI VS RSV 2.5 mg treatment	RSV 10 mg/d VS RSV 2.5 mg/d	12 months	CKMB > 3 UNL
Wang [13]	62/63	Statin naïve	NSTE-ACS	RSV 20 mg before PCI VS placebo pretreatment	RSV 10 mg/d for at least 30 days	30 days	CKMB > 3 UNL
Xie [8]	79/80	Statin naïve	NSTE-ACS	RSV 20 mg 12 h and 20 mg 2 h before PCI VS placebo treatment	RSV 10 mg/d	30 days	cTnI > 5 UNL
Yun [20]	225/ 220	Statin naïve	NSTE-ACS	RSV 40 mg before PCI VS no statin pretreament	RSV 10 mg/d	12 months	CKMB > 2 UNL
Veselka [9]	220/ 225	Mixed	Stable Angina	RSV 20 mg 12 h and 20 mg before PCI VS no statin pretreatment	Statin treatment	In hospital	cTnI > 5 UNL
Cay [21]	153/ 146	Statin naïve	Stable Angina	RSV 40 mg 24 h before PCI VS no RSV pretreatment	RSV 10-40 mg/d	In hospital	CKMB > 3 UNL
Leoncini [11]	252/ 252	Statin naïve	NSTE-ACS	RSV 40 mg 24 h and 20 mg before PCI VS no statin pretreatment	RSV 20 mg/d VS atorvastatin 40 mg/d	6 months	CKMB > 3 UNL
ROMA [16]	80/80	Statin naïve	Stable Angina	RSV 40 mg 24 h before PCI VS no RSV pretreatment	RSV 20 mg/d	12 months	CKMB > 3 UUNL
ROMAII [15]	175/ 100	prior statin treatment	Stable Angina	RSV 40 mg 24 h before PCI VS no statin pretreatment	RSV 20 mg/d	12 months	CKMB > 3 UNL
Ko [12]	62/70	Mixed	STEMI	RSV 40 mg before PCI VS placebo treatment	RSV 40 mg VS 10 mg both for 7 days, and a further 10 mg/d in both groups for 3 weeks	3 months	NA
Liang [10]	66/66	Statin naïve	ACS	RSV 40 mg 4 h VS RSV 10 mg before PCI	RSV 10 mg/d for at least 1 year	6 months	NA

*RSV* rosuvastatin, *CK-MB* creatine kinase-myocardial band, *cTnI* cardiac troponin I, *UNL* upper normal limit, *Mixed* Statin naïve and prior statin treatment, *PMI* periprocedural myocardial infarction, *ACS* acute coronary syndrome, *NSTE-ACS* non-ST segment elevation ACS, *STEMI* ST segment elevation myocardial infarction, *NA* not available

**Table 2:** Baseline clinical characteristics and procedural details in the overall population

Variables	High-dose of RSV n/population (%)	Control n/population (%)
Number of patients	1671/3273 (51.1 %)	1602/3273 (48.9 %)
Male	1150/1671 (68.8 %)	1075/1602 (67.1 %)
Hypertension	1088/1671 (65.1 %)	988/1602 (61.7 %)
Diabetes Mellitus	473/1671 (28.3 %)	449/1602 (28.0 %)
Smoker	592/1606 (36.9 %)	573/1542 (37.2 %)
Previous MI	223/1067 (20.9 %)	224/1069 (21.0 %)
Previous PCI	160/1019 (15.7 %)	143/1017 (14.1 %)
Stable angina	732/1671 (43.8 %)	657/1602 (41.0 %)
NSTEMI	759/1671 (45.4 %)	759/1602 (47.3 %)
STEMI	181/1671 (10.8 %)	188/1602 (11.7 %)
Multi-vessel disease	198/644 (30.7 %)	157/633 (24.8 %)
B2/C lesions	654/918 (71.2 %)	590/855 (69.0 %)
Thrombus	122/757 (16.1 %)	135/763 (17.7 %)
LM	16/700 (2.3 %)	25/709 (3.5 %)
LAD	666/1253 (53.2 %)	619/1174 (52.7 %)
LCX	360/1315 (27.3 %)	354/1244 (28.4 %)
RCA	393/1253 (31.4 %)	368/1174 (31.3 %)
Multiple vessel PCI	216/644 (33.5 %)	203/768 (26.4 %)
Aspirin	1624/1671 (97.2 %)	1548/1602 (96.6 %)
Clopidogrel	1599/1605 (99.6 %)	1530/1536 (99.6 %)
β-blockers	952/1393 (68.3 %)	979/1402 (69.8 %)
ACEI/ARB	910/1393 (65.3 %)	953/1402 (67.9 %)
Glycoprotein IIb/IIIa inhibitors	76/1025 (7.4 %)	103/959 (10.7 %)
Numbers of DES	560/696 (80.5 %)	556/691 (80.5 %)

*Previous MI* previous myocardial infarction, *Previous PCI* previous percutaneous coronary intervention, *NSTE-ACS* non-ST segment elevation acute coronary syndrome, *STEMI* ST segment elevation myocardial infarction, *LM*, left main, *LAD* left anterior descending, *LCX* left circumflex, *RCA* right coronary artery, *DES* drug-eluting stent

**Fig. 2:**
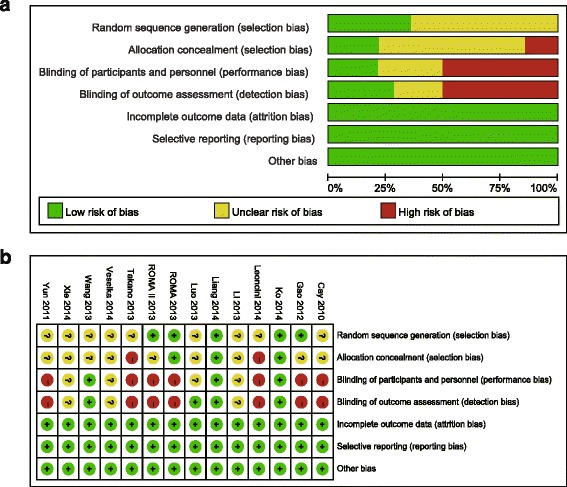
Quality assessment of included studies in this review **a**. Risk of bias graph; **b**. Risk of bias summary

Judgments on each risk of bias item for all studies are shown in Fig. 2. Five of fourteen trials [10, 12, 15, 16, 19] reported the specific methods used for randomization, while the remaining included trials [8, 9, 11, 13, 14, 17, 18, 20, 21] did not mention that. Three trials [10, 12, 15] described the allocation concealments in detail, two trials [11, 14] did not use allocation concealments, and allocation concealments were not described in the remaining trials.[8, 9, 13, 16–21] Blinded methods were used in four trials, three of which [10, 12, 13] used a blinded approach for investigators, participants and outcome assessors, while one trial [17] was only blinded for outcome assessors. None of the included studies had incomplete outcome data, selective reporting and other sources of bias.

### Effect of high-dose RSV preloading before PCI on coronary perfusion

**Fig. 3:**
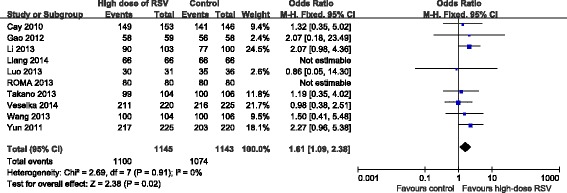
ORs for final TIMI flow grade in overall population. Abbreviations: CI, confidence interval; M-H, Mantel-Haenszel

Effect of high-dose RSV preloading before PCI on post-PCI TIMI flow grade 3 were analyzed in ten trials [9, 10, 13–15, 17–21]. Fixed effect model was chosen based on no potential heterogeneity across trials (*I*^2^ = 0 %, *P* = 0.91). The overall outcome indicated that RSV preloading lead to a 61 % relative increase in post-PCI TIMI flow grade 3 (OR = 1.61, 95 % CI: 1.09-2.38, *P* = 0.02; Fig. 3).

### Effect of high-dose RSV preloading before PCI on clinical outcomes

**Fig. 4:**
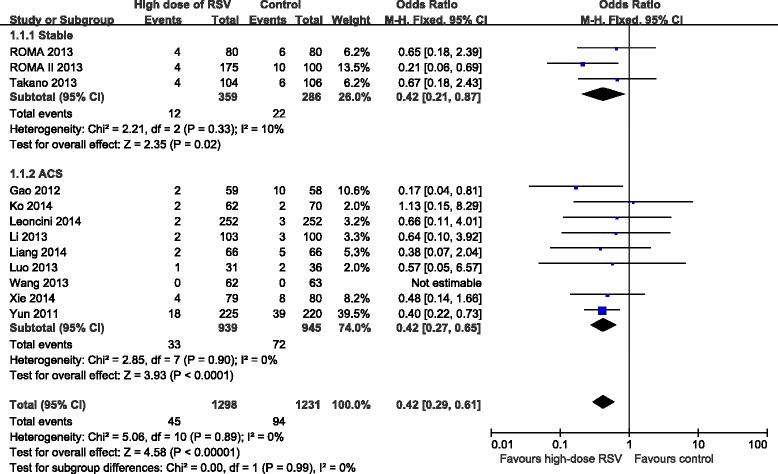
ORs for MACE in patients with different coronary syndromes. Abbreviations: CI, confidence interval; M-H, Mantel-Haenszel

**Fig. 5:**
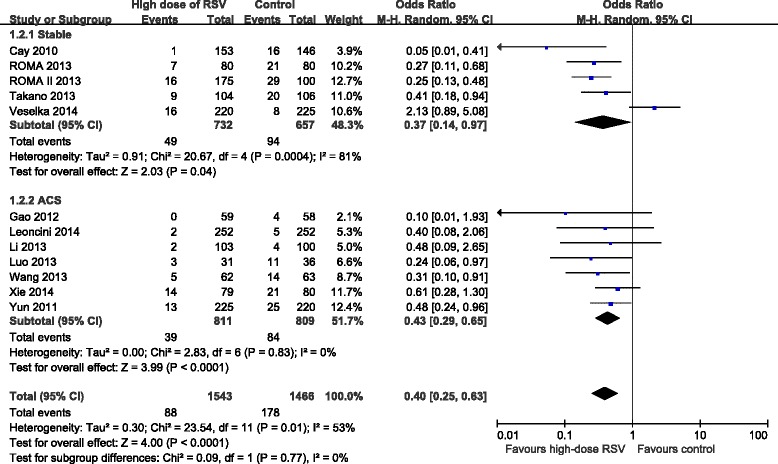
ORs for PMI in patients with different coronary syndromes. Abbreviations: CI, confidence interval; M-H, Mantel-Haenszel

Effect of high-dose RSV preloading before PCI on MACE were analyzed in 12 trials [8, 10–20]. Fixed effect model was chosen based on no potential heterogeneity across trials (*I*^2^ = 0 %, *P* = 0.89). The overall outcome for MACE showed that RSV preloading lead to a 58 % relative reduction in MACE (OR = 0.42, 95 % CI: 0.29-0.61, *P* < 0.00001; Fig. 4). Effect of RSV preloading before PCI on PMI were analyzed in 12 trials [8, 9, 11, 13–21]. Random effects model was used due to substantial heterogeneity between the two groups (*I*^2^ = 53 %, *P* = 0.01). The overall outcome for PMI showed that RSV preloading lead to a 60 % relative reduction in PMI (OR = 0.40, 95 % CI: 0.25-0.63, *P* < 0.0001; Fig. 5).

### Subgroup analyses with different types of coronary syndrome

In order to assess the effect of high-dose RSV preloading in patients with different disease status before undergoing PCI, we divided all enrolled patients into two groups: stable angina and ACS. Due to the fixed effect model, RSV preloading before PCI lead to a 58 % relative reduction in MACE for stable angina patients (OR = 0.42, 95 % CI: 0.21-0.87, *P* = 0.02; *I*^2^ = 10 %); and a 58 % relative reduction in MACE for ACS patients (OR = 0.42, 95 % CI: 0.27-0.65, *P* < 0.0001; *I*^2^ = 0 %) (Fig. 4). We also evaluated the stable angina and ACS subgroup for their effects on PMI. Due to the random effects model, RSV preloading before PCI lead to a 63 % reduction in PMI for stable angina patients (OR = 0.37, 95 % CI: 0.14-0.97, *P* = 0.04; *I*^2^ = 81 %); and a 57 % reduction in PMI for ACS patients (OR = 0.43, 95 % CI: 0.29-0.65, *P* < 0.0001; *I*^2^ = 0 %) (Fig. 5).

### Subgroup analyses based on previous statin therapy

**Fig. 6:**
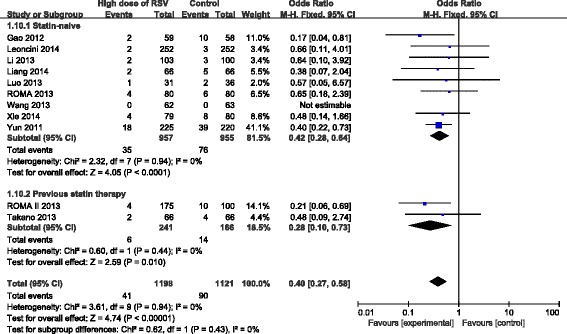
ORs for MACE in patients with different statin therapy. Abbreviations: CI, confidence interval; M-H, Mantel-Haenszel

**Fig. 7:**
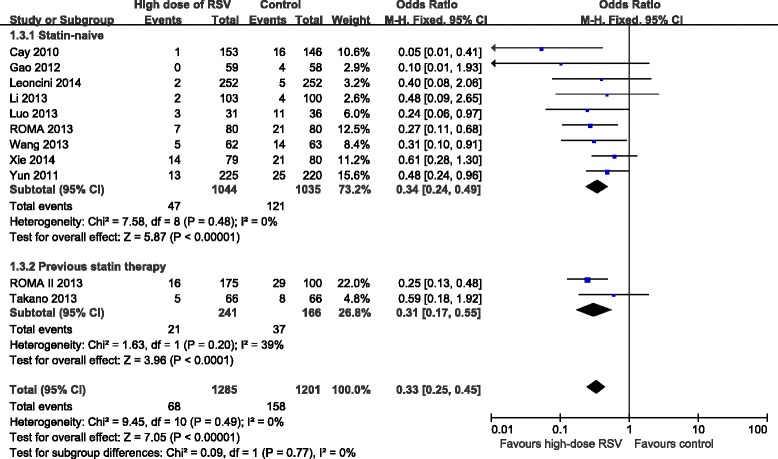
ORs for PMI in patients with different statin therapy. Abbreviations: CI, confidence interval; M-H, Mantel-Haenszel

In order to confirm whether current statin therapy before high-dose RSV preloading prior to PCI affected the incidence of MACE and PMI, we evaluated subgroups of patients based on their statin therapy before PCI in 11 trials [8, 10, 11, 13–20]. Due to the fixed effect model, RSV preloading lead to a 60 % relative reduction of MACE in all the enrolled patients (OR = 0.40, 95 % CI: 0.27-0.58, *P* < 0.00001; *I*^2^ = 0 %), a 58 % relative reduction in the statin naïve group (OR = 0.42, 95 % CI: 0.28-0.64, *P* < 0.0001; *I*^2^ = 0 %) and a 72 % relative reduction in the previous statin therapy group (OR = 0.28, 95 % CI: 0.10-0.73, *P* = 0.01; *I*^2^ = 0 %) (Fig. 6). Due to the fixed effect model, RSV preloading lead to a 67 % relative reduction of PMI in all the enrolled patients (OR = 0.33, 95 % CI: 0.25-0.45, *P* < 0.00001; *I*^2^ = 0 %), a 66 % relative reduction in the statin naïve group (OR = 0.34, 95 % CI: 0.24-0.49, *P* < 0.00001; *I*^2^ = 0 %), and a 69 % relative reduction in the previous statin therapy group (OR = 0.31, 95 % CI: 0.17-0.55, *P* < 0.0001; *I*^2^ = 39 %) (Fig. 7).

### Effects of high-dose RSV preloading on follow-up outcome

**Table**3 depicts overall MACE including mortality, spontaneous MI, and TVR during follow-up. As mentioned above, the overall MACE was significantly reduced in all the enrolled patients. When stratified by type of disease, high-dose RSV preloading before PCI failed to decrease overall mortality (*P* = 0.42), spontaneous MI (*P* = 0.34) and TVR (*P* = 0.05) in patients with stable angina, and also didn’t reduce overall mortality in ACS patients (*P* = 0.09). However, RSV preloading before PCI significantly reduced the incidence of spontaneous MI (*P* = 0.02) and TVR (*P* = 0.01) in ACS patients. According to previous statin therapy, high-dose RSV preloading had no influence on overall mortality (*P* = 0.3), spontaneous MI (*P* = 0.13) and TVR (*P* = 0.09) in previous statin therapy patients. Moreover, the overall mortality (*P* = 0.09) was not significantly reduced in statin naïve patients, while the incidence of spontaneous MI (*P* = 0.02) and TVR (*P* = 0.008) was significantly decreased.

**Table 3:** Clinical events during follow-up

Events	High-dose of RSV n (%)	Control n (%)	P
Stable			
Death	2(0.6 %)	3(1.0 %)	0.42
Spontaneous MI	5(1.4 %)	7(2.4 %)	0.34
TVR	5(1.4 %)	12(4.2 %)	0.05
MACE	12(3.3 %)	22(7.7 %)	0.02
ACS			
Death	7(0.7 %)	15(1.6 %)	0.09
Spontaneous MI	7(0.7 %)	20(2.1 %)	0.02
TVR	19(2.0 %)	37(3.9 %)	0.01
MACE	33(3.5 %)	72(7.6 %)	<0.0001
Overall			
Death	9(0.7 %)	18(1.5 %)	0.06
Spontaneous MI	12(0.9 %)	27(2.2 %)	0.01
TVR	24(1.8 %)	49(4.0 %)	0.002
MACE	45(3.5 %)	94(7.6 %)	<0.00001
Statin naïve			
Death	7(0.7 %)	15(1.6 %)	0.09
Spontaneous MI	8(0.8 %)	21(2.2 %)	0.02
TVR	20(2.1 %)	40(4.2 %)	0.008
MACE	35(3.7 %)	76(8.0 %)	<0.0001
Prior statin treatment			
Death	1(0.4 %)	2(1.2 %)	0.3
Spontaneous MI	2(0.8 %)	5(3.0 %)	0.13
TVR	3(1.2 %)	7(4.2 %)	0.09
MACE	6(2.4 %)	14(8.4 %)	0.01
Overall			
Death	8(0.7 %)	17(1.5 %)	0.05
Spontaneous MI	10(0.8 %)	26(2.3 %)	0.005
TVR	23(1.9 %)	47(4.2 %)	0.002
MACE	41(3.4 %)	90(8.0 %)	<0.00001

*ACS* acute coronary syndrome, *Spontaneous MI* spontaneous myocardial infarction, *TVR* target vessel revascularization, *MACE* major adverse cardiovascular events

### Publication bias

**Fig. 8:**
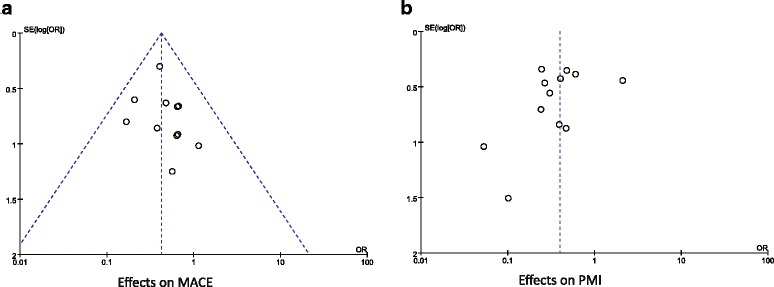
Funnel plots of the included studies. **a**. Funnel plots for MACE; **b**. Funnel plots for PMI. The results show no potential publication bias for MACE and PMI. Abbreviations: OR, odds ratio; SE, standard error

As shown in Fig. 8, the results didn’t provide any evidence of potential publication bias based on funnel plots and Egger’s regression test. Funnel plots for MACE were generated using a fixed effect model (*P* = 0.139 > 0.05) and for PMI were generated using a random effects model (*P* = 0.273 > 0.05).

## Discussion

Our analysis of 3273 individuals from 14 RCTs demonstated that high-dose RSV preloading before PCI can significantly improve the post-PCI TIMI flow grade and reduce the incidence of MACE and PMI. In addition, according to different disease presentations, high-dose RSV preloading can significantly decrease the incidence of MACE and PMI in both stable angina and ACS patients; when stratified according to current statin therapy, high-dose RSV preloading can also significantly reduce the incidence of MACE and PMI in both statin naïve and previous statin therapy patients. Moreover, according to the follow-up data, high-dose RSV preloading can significantly reduce the incidence of spontaneous MI and TVR in ACS and statin naïve patients, but failed to improve clinical outcomes in both patients with stable angina and current statin therapy.

We confirm and extend the meta-analyses by Benjo A.M [22] and Wang L [7] which indicated a reduction in PMI and MACE with statin preloading before PCI. Benjo *et al.* selected 1591 patients from 14 RCTs and found a 56 % relative reduction in PMI and a 41 % reduction in clinical events in patients with current statin treatment before PCI. Wang L *et al.* selected 24 RCTs with 5526 patients and demonstrated that statin preloading lead to a 59 % relative reduction in PMI and a 39 % relative reduction in MACE. These two studies were credible and conclusive, while different types of statins may have various effects on clinical events in both studies. Although the former study was powerful and proved their hypothesis, patients with STEMI and current statin treatment were excluded, which led to the lack of identifying the effects of statin preloading in the overall population. Data on patients with chronic statin therapy were included in the latter study, however, recent trials on high-dose RSV pretreatment, which were published after this meta-analysis, drew controversial conclusions concerning the cardioprotective benefits of this treatment on PMI and MACE. Our meta-analysis demonstrated that RSV preloading before PCI lead to a 60 % relative reduction in PMI (*P* < 0.0001) and a 58 % relative reduction in MACE (*P* < 0.00001). The cardioprotective effects of RSV were consistent in not only stable angina and ACS patients but also statin naïve and previous statin therapy patients. High-dose RSV preloading can significantly reduce the clinical outcome in the overall population, which was mainly due to a reduction of TVR, especially for ACS and statin naïve patients. Although these benefits of RSV preloading failed to decrease overall mortality in the enrolled patients, they reduced the incidence of spontaneous MI and TVR in ACS and statin naïve patients.

PMI is characterized by any cardiac serum marker (creatine kinase-myocardial band [CK-MB]; cardiac troponin I [cTnI]) elevation three times or more the upper limit of normal (ULN) after PCI, which has been shown to occur in approximately 15-20 % of patients undergoing PCI procedures in spite of different definitions used [23]. The pathogenic mechanisms of PMI include side-branch occlusion, distal embolism, endothelial dysfunction, oxidative stress, and inflammation [1]. In order to reduce the incidence of PMI, researchers have focused on antiplatelet agents, anticoagulants, vasodilators, and beta-blockers to improve coronary blood flow after PCI. More recently, some studies [21, 24, 25] have suggested that the reduction of the MACE and PMI in patients undergoing PCI was associated with HMG-CoA reductase inhibitors (statins), in particular atorvastatin and RSV. In addition to its lipid-lowering effects, RSV exerts many cardioprotective effects including an improvement in endothelial function, antithrombotic actions, inhibition of thrombosis, plaque stabilization, and suppression of inflammation. These may partly account for the cardioprotective effects of RSV on spontaneous MI and TVR in ACS patients due to the high inflammatory status in these patients, which is associated with a high predictive value for the occurrence of MACE [26].

The possible mechanisms underlying the early protective action of RSV are unclear, as the enrolled patients were given a short-term high-dose of RSV before PCI, which may not have had a significant influence on cholesterol level. Vilahur G *et al.* [27] showed that RSV enhanced PKC, Erk2, AKT/PKB signal pathways and its downstream effectors to attenuate inflammation and cardiomyocyte apoptosis in the peri-infarcted zone and reduce infarct size in pigs. RSV has been shown to enhance the protective effects of ischemic post-conditioning against myocardial ischemia and reperfusion (I/R) injury in rats via activating PI3K/Akt/eNOS signaling pathway [28]. Nitrous oxide (NO), due to the activation of endothelial nitric oxide synthase (eNOS), can decrease leukocyte activation and infiltration, platelet activation and aggregation, vasoconstriction and contractile dysfunction [1]. These beneficial cardiac effects of statins are absent in eNOS knockout mice and can be reversed by using the specific inhibitor of PI3K kinase and eNOS [29, 30]. In our meta-analysis, cardiac benefits of high-dose RSV preloading before PCI decreased the incidence of spontaneous MI and TVR in statin naïve patients, but had no effects on previous statin therapy patients. Patients with previous statin therapy are probably in a condition of dyslipidaemia. They are likely to take some nutraceuticals (resveratrol, grape seed, curcumin, zinc, and fish oil) and antioxidants (carotenoids, vitamins A, C, and E) in their daily diet to decrease the plasma lipids, which can influence the overall outcomes [31, 32]. Hence, we speculate that the cardiovascular benefits of short-term high-dose RSV preloading might be counteracted by long-term intake of statin, nutraceuticals, or antioxidants. Interestingly, chronic statin therapy failed to exert a cardioprotective effect that wanes with time associated with increased levels of PTEN (phosphatase and tensin homolog deleted on chromosome ten, an inhibitor of PI3K) in SD rats and these can partly attenuate the cardioprotective effects of high-dose RSV preloading, which may partially confirm our speculation [33].

The results of our study are different from those by Veselka *et al.* [9], who demonstrated that high-dose RSV therapy had no effects on the incidence of PMI in patients with stable angina. We compared the results when included and excluded Veselka’s trial data. The significant effects on PMI were unchanged, while excluding Veselka’s data changed the homogeneity in the stable angina group (*I*^2^ = 81 to 16 %) and the overall population (*I*^2^ = 53 to 0 %). We indicated that the main heterogeneity was due to the different doses and types of statins administered. 36 of 220 patients (16.4 %) in the high-dose RSV group and 51 of 225 patients (22.6 %) in control received long-term high-dose statin therapy (atorvastatin 40 or 80 mg, RSV 20 or 40 mg). This may partially explain why high-dose RSV treatment before PCI failed to reduce the incidence of PMI in their study, as the beneficial cardioprotection effect of transient high-dose RSV may be eliminated by chronic high-dose statin therapy.

Statin therapy is only recommended as secondary prevention of cardiovascular outcomes in the present guidelines for ACS and PCI [34, 35]. However, high-dose RSV preloading before PCI can reduce the incidence of MACE and PMI, which is not recommended in these guidelines. Our analysis adds strength and power to current recommendations and potentially expands the use of RSV before PCI.

### Study limitations

There were several limitations in our meta-analysis. Firstly, the trials included did not use a uniform RSV regimen, definition of PMI, or clinical outcomes examined. Different doses and duration of RSV treatment in patients with different backgrounds may have various effects on MACE and PMI. Secondly, seven of fourteen trials included not long enough follow-up period which was no more than 30 days of observation. Hence, more high quality RCTs are required to identify the beneficial cardiac effects of RSV preloading before PCI over a longer follow-up period. Thirdly, due to a lack of patient-level data, we failed to analyze the effect of high-dose RSV preloading on peri-procedural high-sensitivity C-reactive protein (hs-CRP) level variation (post-intervention hs-CRP minus baseline hs-CRP). In the same way, we are not able to provide subgroup analyses based on gender due to a lack of patient-level data. Hence, further studies are required to investigate whether high-dose RSV preloading plays a different role in men and women. Finally, there was potential heterogeneity in our study due to limited study numbers, small sample sizes, different protocols, and patients with various backgrounds.

## Conclusion

High-dose RSV preloading can significantly improve myocardial perfusion and reduce MACE and PMI in patients undergoing PCI. The cardioprotective effects of RSV preloading were significant in not only stable angina and ACS patients but also statin naïve and previous statin therapy patients. The cardioprotective effects of high-dose RSV were mainly due to the reduction in spontaneous MI and TVR, especially in ACS and statin naïve patients. Therefore, it indicates that RSV preloading before PCI should be used in consideration of the disease presentation and current statin therapy in patients before undergoing PCI.

## Abbreviations

RSV: Rosuvastatin
PCI: Percutaneous coronary intervention
PMI: Peri-procedural myocardial injury
MACE: Major adverse cardiac events
RCTs: Randomized controlled trials
MI: Myocardial infarction
TVR: Target vessel revascularization
HR: Hazard ratio
OR: Odds ratio
CI: Confidence intervals
ACS: Acute coronary syndrome
HMG-CoA: 3-hydroxy-3-methylglutaryl coenzyme A
TIMI: Thrombolysis in myocardial infarction
hs-CRP: High-sensitivity C-reactive protein
GPIs: Glycoprotein IIb/IIIa inhibitors
ULN: Upper limit of normal
I/R injury: Ischemia and reperfusion injury
NO: Nitrous oxide
eNOS: Nitric oxide synthase
PTEN: Phosphatase and tensin homolog deleted on chromosome ten
CK-MB: Creatine kinase-myocardial band
cTnI: cardiac troponin I
NSTE-ACS: Non-ST segment elevation ACS
STEMI: ST segment elevation myocardial infarction
NA: Not available
LM: Left main
LAD: Left anterior descending
LCX: Left circumflex
RCA: Right coronary artery
DES: Drug-eluting stent
M-H: Mantel-Haenszel
SE: Standard error
